# Parasitism by *Amblyomma rotundatum* on Teiidae lizards in the eastern part of the state of Acre, Brazil

**DOI:** 10.1590/S1984-29612023050

**Published:** 2023-09-01

**Authors:** Simone Delgado Tojal, Ivaneide Nunes da Costa, André de Abreu Rangel Aguirre, Thiago Fernandes Martins, Marcelo Bahia Labruna, Dionatas Ulises de Oliveira Meneguetti, Paulo Sérgio Bernarde, Karoline Silva da Cruz, Jônatas Machado Lima, Sergio Luiz Prolo, Luís Marcelo de Aranha Camargo

**Affiliations:** 1 Programa de Pós-Graduação em Ciências da Saúde, Universidade Federal São João del-Rei – UFSJ, Divinópolis, MG, Brasil; 2 Laboratório de Medicina Tropical, Universidade Federal do Acre – UFAC, Rio Branco, AC, Brasil; 3 Programa de Pós-Graduação em Biologia Parasitária, Fundação Oswaldo Cruz de Rondônia – Fiocruz/RO, Porto Velho, RO, Brasil; 4 Laboratório de Entomologia, Fundação Oswaldo Cruz de Rondônia – Fiocruz/RO, Porto Velho, RO, Brasil; 5 Departamento de Laboratórios Especializados, Superintendência de Controle de Endemias, Secretaria de Estado da Saúde de São Paulo – SUCEN, São Paulo, SP, Brasil; 6 Departamento de Medicina Veterinária Preventiva e Saúde Animal, Universidade de São Paulo – FMVZ/USP, São Paulo, SP, Brasil; 7 Programa de Pós-Graduação em Ciências da Saúde na Amazônia Ocidental, Universidade Federal do Acre – UFAC, Rio Branco, AC, Brasil; 8 Laboratório de Herpetologia, Universidade Federal do Acre – UFAC, Cruzeiro do Sul, AC, Brasil; 9 Laboratório de Ornitologia, Universidade Federal do Acre – UFAC, Rio Branco, AC, Brasil; 10 Programa de Pós-Graduação em Ciência Inovação e Tecnologia para a Amazônia – PPGCITA, Universidade Federal do Acre – UFAC, Rio Branco, Acre, Brasil; 11 Instituto de Ciências Biomédicas, Universidade de São Paulo – ICB5/USP, Monte Negro, RO, Brasil; 12 Instituto Nacional de Epidemiologia da Amazônia Ocidental – INCT-EpiAmO, Porto Velho, RO, Brasil; 13 Centro de Pesquisas em Medicina Tropical – CEPEM, Porto Velho, RO, Brasil; 14 Departamento de Medicina, Centro Universitário FAEMA, RO, Brasil

**Keywords:** Ticks, ectoparasites, Squamata, hosts, Amazonia, Carrapatos, ectoparasitos, Squamata, hospedeiros, Amazônia

## Abstract

The aim of the present study was to report on the occurrence of parasitism by *Amblyomma rotundatum* ticks on two species of Teiidae lizards and test the presence of rickettsiae in the collected ticks, in the western Brazilian Amazon region. Ticks were collected in July 2019, from a fragment of *terra firme* forest in the municipality of Senador Guiomard, Acre, Brazil. Two lizards that were infested by immature stages of ticks were caught using mist net and Tomahawk traps. Ectoparasites were collected manually, and the lizard specimens were identified and released at the same location where they had been caught. Three nymphs and 49 larvae were collected from *Ameiva ameiva*, while 25 nymphs and nine larvae were collected from *Tupinambis cuzcoensis*, which are both in the family Teiidae. The ticks were identified morphologically as belonging to the genus *Amblyomma*. Nymphs were identified at species level through molecular analysis, resulting in the tick species *Amblyomma rotundatum*. This is the first record of parasitism by the tick *A. rotundatum* on *T. cuzcoensis* lizard, and the first report of an association between *A. rotundatum* and the lizard species *A. ameiva* and *T. cuzcoensis* in Acre, in the western part of the Amazon region.

## Resumo

O objetivo do presente estudo foi relatar a ocorrência de parasitismo por carrapatos *Amblyomma rotundatum* em duas espécies de lagartos Teiidae, e testar a presença de riquétsias nos carrapatos coletados, na região da Amazônia Ocidental brasileira. Os carrapatos foram coletados em julho de 2019, em um fragmento de floresta de terra firme no município de Senador Guiomard, Acre, Brasil. Dois lagartos infestados por estágios imaturos de carrapatos foram capturados com rede de neblina e armadilhas *Tomahawk*. Os ectoparasitos foram coletados manualmente, e os espécimes de lagartos foram identificados e soltos no mesmo local onde foram capturados. Três ninfas e 49 larvas foram coletadas de *Ameiva ameiva*, enquanto 25 ninfas e nove larvas foram coletadas de *Tupinambis cuzcoensis*, ambas da família Teiidae. Os carrapatos foram identificados morfologicamente como pertencentes ao gênero *Amblyomma*. As ninfas foram identificadas em nível de espécie por meio de análise molecular, resultando na espécie de carrapato *Amblyomma rotundatum*. Este é o primeiro registro de parasitismo pelo carrapato *A. rotundatum* em lagarto *T. cuzcoensis*, e o primeiro relato de associação entre *A. rotundatum* e as espécies de lagartos *A. ameiva* e *T. cuzcoensis*, no Acre, na parte ocidental da região amazônica.

*Amblyomma rotundatum* Koch, 1844, is an ixodid tick with widespread biogeographical distribution extending from the southern part of the United States to northern Argentina (Labruna et al., 2005a; Guglielmone & Nava, 2010). It is a parthenogenetic species that is known to feed on ectothermic animals (amphibians and reptiles) (Labruna et al., 2004).

Parasitism by *A. rotundatum*, in all its parasitic stages, has been most frequently recorded on frogs of the genus *Rhinella* and on the snake species *Boa constrictor* Linnaeus, 1758 (Guglielmone & Nava, 2010). However, reports of *A. rotundatum* feeding on some mammals have also been published, including accidental findings on humans (Guglielmone & Nava, 2010).

In Brazil, reports of parasitism by *A. rotundatum* on a variety of reptiles are becoming increasingly frequent, with especially to Squamata (Mendoza-Roldan et al., 2020; Souza et al., 2020; Pacheco et al., 2021; Dantas-Torres et al., 2022).

Reports of infestations by *A. rotundatum* on lizards are relatively rare in Brazil. In the Brazilian Amazon region, this parasite has been found feeding on species in the families Teiidae (on *Ameiva ameiva* Linnaeus, 1758, *Kentropyx calcarata* Spix, 1825, *Tupinambis teguixin* Linnaeus, 1758 species) and Tropiduridae (*Plica plica* Linnaeus, 1758, *Uranoscodon superciliosus* Linnaeus, 1758, *Tropidurus* sp.) (Labruna et al., 2005b; Gomides et al., 2015; Zimmermann et al., 2018; Pacheco et al., 2021; Dantas-Torres et al., 2022). About these studies in Amazon, analysing the association of lizard-*A. rotundatum*-microorganism, Dantas-Torres et al. (2022), obtained positive result for bacteria from the genus *Rickettsia* in *A. rotundatum*.

The Brazilian state of Acre forms part of Amazonia, with a variety of ecosystems and habitats, and diversity high in the reptile fauna (Bernarde et al., 2013). Knowledge about ticks that feed on reptiles in this state is limited to recent publications (Mendoza-Roldan et al., 2020; Souza et al., 2020). Moreover, only Souza et al. (2020) has reported parasitism of a lizard species by a tick species of the genus *Amblyomma.* The aim of the present study was to report on the occurrence of parasitism by *A. rotundatum* ticks on two species of Teiidae lizards and test the presence of rickettsiae in the collected ticks, in the eastern part of the state of Acre, in the western Brazilian Amazon region.

**Figure 1:**
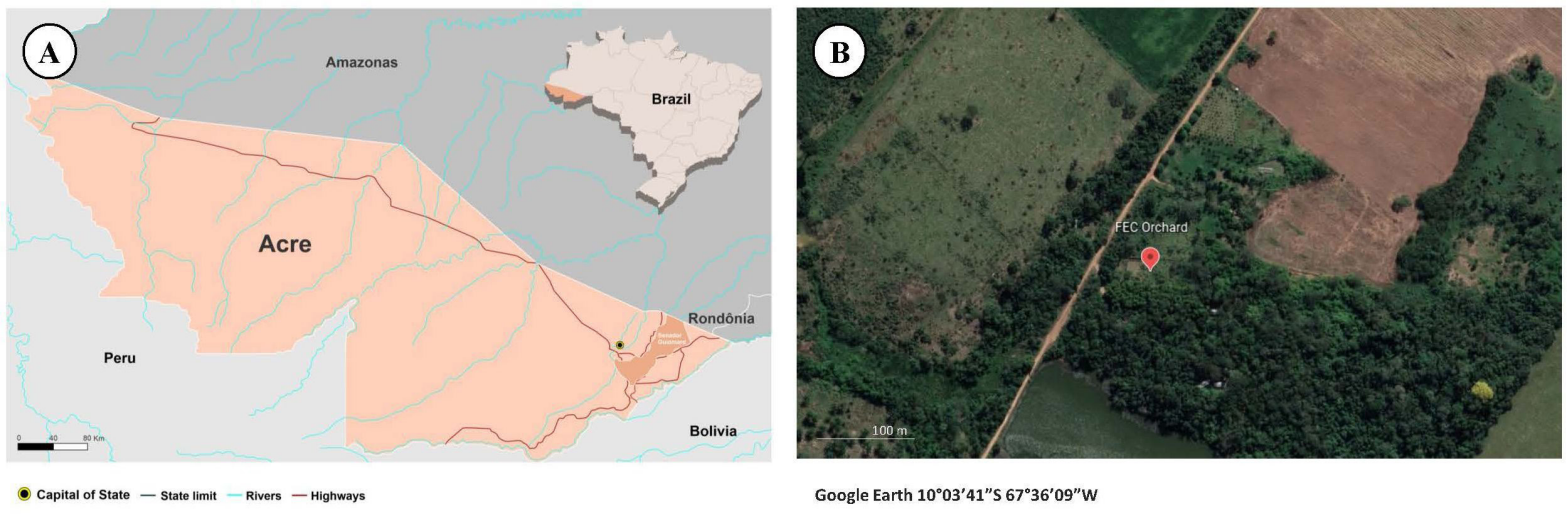
(A) Map showing the location of the municipality of Senador Guiomard, Acre, Brazil; (B) Location of the collection area.

**Figure 2:**
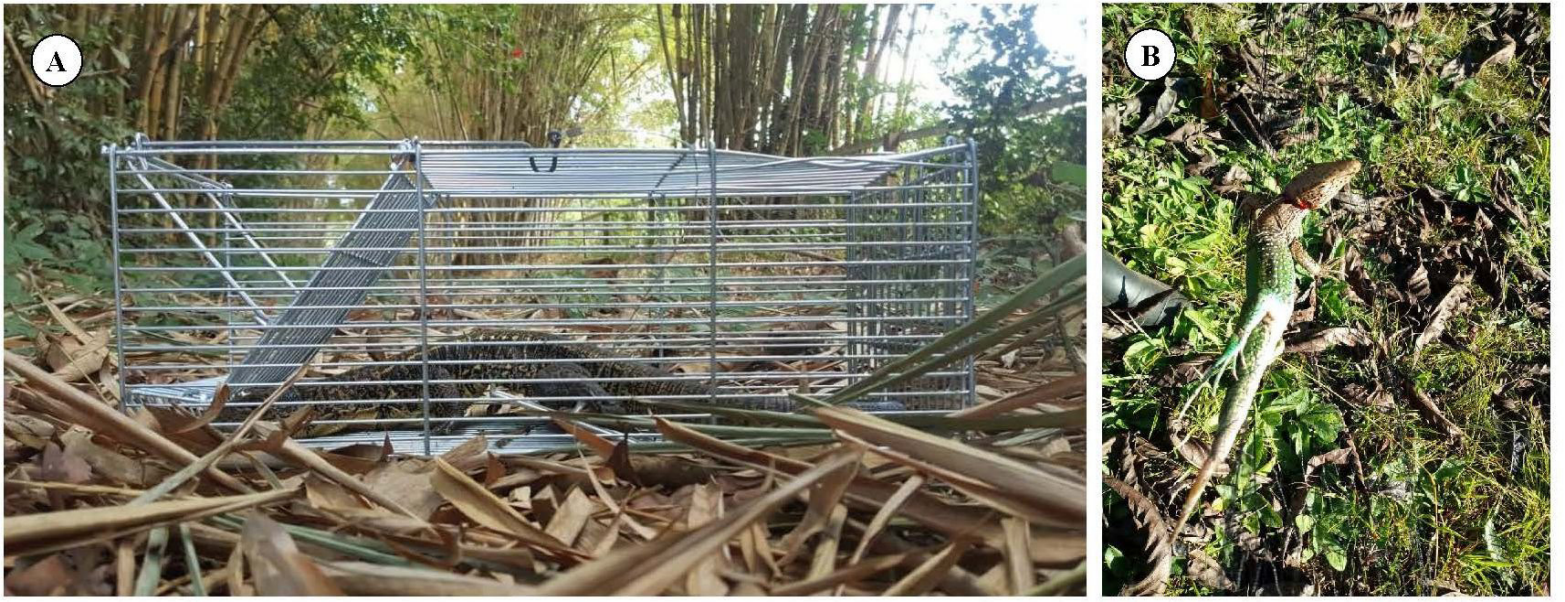
(A) *Tupinambis cuzcoensis* lizard; (B) *Ameiva ameiva* lizard.

Tick collection was performed on July 12, 2019, in a reserve belonging to the Federal University of Acre (Catuaba Experimental Farm: 10°03’41” S; 67°36’09” W). This area consists of a fragment of *terra firme* forest, located in the municipality of Senador Guiomard, in the eastern part of the state of Acre, Brazil (Silveira et al., 2020) (Figure 1). The hosts were caught in traps that are unusual for this taxon: one lizard (*A. ameiva*) was caught in a mist net and the other (*Tupinambis cuzcoensis*) in a Tomahawk trap (Figure 2). *Ameiva ameiva* lizard was being preyed on by a bird-of-prey that bumped into the mist net, releasing its prey into it. Both lizards were infested by immature stages of ticks, which were collected manually using tweezers and placed inside plastic tubes containing 70% alcohol (ethanol). The lizards were identified using a guide and a recent paper (Vitt & Zani, 1998; Silva et al., 2018) and then released at the same capture site. The specimens were collected under authorization from the Chico Mendes Institute for Biodiversity Conservation: SisBio license number 69.943-4.

The ticks thus collected were transported to the laboratory of the School of Veterinary Medicine and Animal Science of the University of São Paulo for morphological examination with the aid of a stereoscopic magnifying glass and a taxonomic key for nymphs (Martins et al., 2010) and larvae by specialist. Subsequently, larvae and nymphs were taken to the Entomology Laboratory of the Oswaldo Cruz Foundation of Rondônia for molecular analysis.

Tick DNA was extracted using the guanidinium isothiocyanate and phenol/chloroform (Sangioni et al., 2005). The extracted DNA was stored at Fiocruz Rondônia, Porto Velho, Rondônia state. For molecular identification of tick species, the DNA samples were submitted to Polymerase Chain Reaction (PCR) to amplify fragments of three genes: one from 16S mitochondrial rDNA of 460 bp (Mangold et al., 1998), another fragment of 649 bp from ribossomal Internal Transcribed Spacer 2 (ITS2) gene, using ITS-2F e ITS-2R primers (Csordas et al., 2016), and other fragment of 658 bp of the mitochondrial gene cytochrome oxidase I (COI) (Folmer et al., 1994). Other PCR assays were performed to analyze the presence of *Rickettsia* DNA, with PCRs to amplify a 401-bp fragment of the citrate synthase gene (*gltA*), which is present in all species of *Rickettsia* (Labruna et al., 2004).

PCR products with specific bands on agarose gels were purified using ExoSAP-ITTM (Thermo Fisher Scientific, USA) and were sequenced on the DNA sequencing platform of the René Rachou Institute, Fiocruz Minas Gerais, Fundação Oswaldo Cruz, Brazil.

The Sanger method (Sanger et al., 1977) was applied, in replicate, using an ABI 3730xL sequencer (Applied Biosystems, USA). A phylogenetic tree was constructed to compare the nucleotide sequences from ticks, with the sequences of *Amblyomma* spp. in GenBank. The evolutionary history was inferred by using the Maximum Likelihood method and Tamura 3-parameter model (Tamura, 1992). The bootstrap consensus tree inferred from 500 replicates is taken to represent the evolutionary history of the taxa analyzed. Branches corresponding to partitions reproduced in less than 50% bootstrap replicates are collapsed. The percentage of replicate trees in which the associated taxa clustered together in the bootstrap test (500 replicates) are shown next to the branches. Initial tree(s) for the heuristic search were obtained automatically by applying Neighbor-Join and BioNJ algorithms to a matrix of pairwise distances estimated using the Maximum Composite Likelihood (MCL) approach, and then selecting the topology with superior log likelihood value. A discrete Gamma distribution was used to model evolutionary rate differences among sites (5 categories (+G, parameter = 0.2085)). This analysis involved 17 nucleotide sequences. All positions containing gaps and missing data were eliminated (complete deletion option). There was a total of 398 positions in the final dataset. Evolutionary analyses were conducted in MEGA X (Kumar et al., 2018).

Eighty-six ticks were collected from the lizards: three nymphs (N) and 49 larvae (L) were collected from *A. ameiva* (Linnaeus, 1758), while 25 nymphs and nine larvae were collected from *T. cuzcoensis* Murphy, Jowers, Lehtinen, Charles, Colli, Peres, Hendry & Pyron, 2016. Both of these lizards belong to the family Teiidae (Squamata, Sauria). All the ticks were morphologically identified as belonging to the genus *Amblyomma*.

Individual nymphs (3 N found on *A. ameiva*, 25 N on *T. cuzcoensis*) and pools containing 9-10 larval specimens (5 pool-L on *A. ameiva*, 1 pool-L on *T. cuzcoensis*) (n = 34) were sent for amplification of the COI gene. However, only 26 PCR products from the fragment of this gene were observed on the agarose gel.

**Figure 3:**
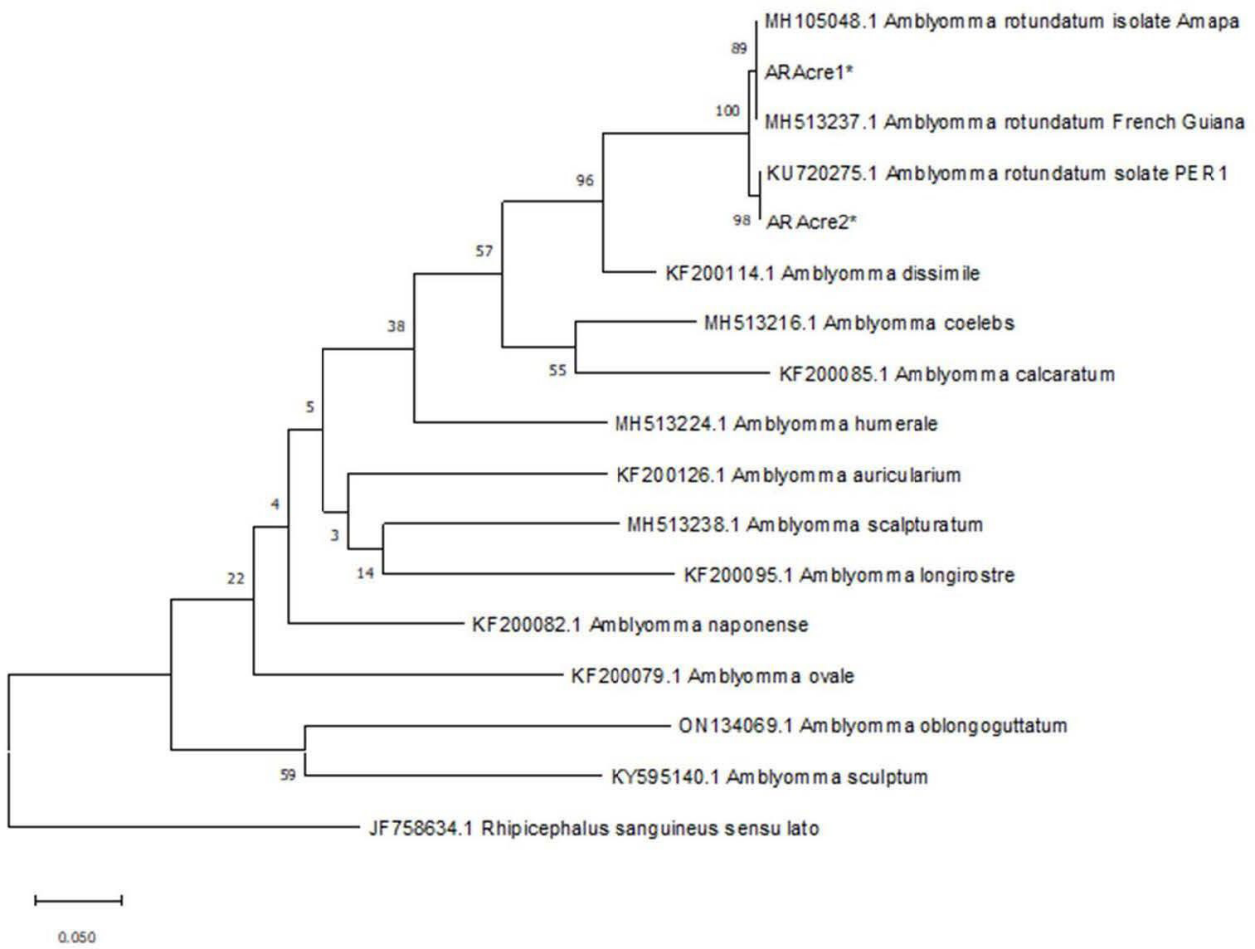
Phylogenetic tree of the *Amblyomma rotundatum* partial sequences ARAcre1 and ARAcre2. The maximum likelihood method was used to infer the evolutionary history of the COI sequences of this study and other sequences found in GenBank.

A total of 19 sequences were identical to *A. rotundatum* partial COI sequences. The other 7 sequences obtained were of poor quality and could not be compared with other COI sequences in GenBank. Among the 19 sequences, 13 presented a sequence of 658 bp that was 100% identical to *A. rotundatum* isolates from the state of Amapá, in the eastern Brazilian Amazon region. These 13 sequences were named here as ARAcre1 (GenBank: MH105048.1): three larval pools (10 specimens each) and two nymphs of *A. ameiva*; and eight nymphs of *T. cuzcoensis*. The other 6 sequences were 100% identical to *A. rotundatum* isolates from Peru (western Amazon region), named here as ARAcre2 (GenBank: KU720275.1): six nymphs of *T. cuzcoensis* (Figure 3). The ARAcre1 and ARAcre2 sequences are 4 single nucleotide polymorphisms (SNPs) from the consensus sequence of the Peru isolates. No amplification of DNA from *Rickettsia* spp. was observed on the agarose gel, from the tick samples analyzed.

According to the literature, *A. rotundatum* seems to be a species that is widely distributed in Brazil. Its presence has been reported in all regions of the country, encompassing the states of Acre, Amazonas, Rondônia, Goiás, Mato Grosso, Mato Grosso do Sul, Minas Gerais, Pará, Paraná, Maranhão, Pernambuco, Ceará, Rio Grande do Sul, Rio de Janeiro, Santa Catarina and São Paulo (Labruna et al., 2005a; Polo et al., 2021).

Recently, a distribution model for the populations of *A. rotundatum* and *A. dissimile* was constructed by Polo et al. (2021). They demonstrated that the species *A. rotundatum* presented greater aptitude for dispersion in all of Brazil’s biomes, whereas *A. dissimile* was restricted to the Amazon and Pantanal biomes. From this perspective of the distribution of *A. rotundatum and A. dissimile*, which are close-related tick species, the importance of our study is that it provides molecular confirmation that nymphs of *A. rotundatum* were found in the southwestern Amazon region. It is noteworthy that although we have molecularly detected *A. rotundatum* larvae parasitizing *A. ameiva*, it is not possible to affirm that all the thirty larvae belong to this tick species, because the PCRs were performed in three pools of larvae DNA, and larvae were not tested individually.

Regarding the hosts that were found, Teiidae are diurnal terrestrial lizards that are commonly encountered foraging in cleared and transitional areas of the Amazon Forest, Cerrado and Caatinga (Vitt & Zani, 1998). The species of this study, popularly known in the region as "calango" (*A. ameiva*) and "teju-açú" (*T. cuzcoensis*), were found close to areas that had been altered for crop cultivation. Parasitism by *A. rotundatum* in the Teiidae species *A. ameiva* in the Neotropical region (Guglielmone & Nava, 2010), including in Brazil, is already known, with reports from the Amazon biome (Dantas-Torres et al., 2022) and from a sandbar terrain (*restinga*), on the coast of the state of Rio de Janeiro (Viana et al., 2012). Silva et al. (2018) provided a description of a new species of lizard of the genus *Tupinambis* in the central region of South America and presented a new distribution map for the species of the genus *Tupinambis*. They demonstrated that the only species of this genus with distribution in Acre is *T. cuzcoensis*, and not *T. teguixin*, how it was described. Thus, the present study provides the first record on the tick-host relationship between *A. rotundatum* and the Teiidae species *T. cuzcoensis* in the Amazon, as well as the first report on the association between *A. rotundatum* and both of these Teiidae lizards (*A. ameiva* and *T. cuzcoensis*) in the state of Acre.

The findings regarding *T. cuzcoensis* nymphs are also of interest because the specimen examined here was parasitized with two distinct COI partial sequences of *A. rotundatum,* which had previously only been found in two separate localities in the Amazon region, (state of Amapá, in the eastern Amazon region of northern Brazil; and Peru; in the western Amazon region). This question is one of the limitations of the present study because answering it requires more robust analyses, including the thorough analysis of other genes, such as 12S rRNA, 16S rRNA and ITS2. Here, we can propose further investigations on the identification of distinct genotypes of this tick species and the limiting factors of its populations.

This study is the first record of parasitism by *A. rotundatum* tick on *T. cuzcoensis* lizard, a Teiidae lizard species, and the first report on the association between *A. rotundatum* and both of the lizard species *A. ameiva* and *T. cuzcoensis* in Acre, in the western Brazilian Amazon region. Considering that studies on ticks in association with reptiles in the Amazon region are uncommon, the present study adds knowledge about the biology and ecology of this tick species, for which lifecycle and preferred-host information is sparse.
